# CPGminer: An Interactive Dashboard to Explore the Genomic Features and Taxonomy of Complete Prokaryotic Genomes

**DOI:** 10.3390/microorganisms11102556

**Published:** 2023-10-13

**Authors:** Jaehyun Kim, Sunghyun Yoon, Sandeep Kondakala, Steven L. Foley, Mark Hart, Dong-Heon Baek, Wenjun Wang, Sung-Kwan Kim, John B. Sutherland, Seong-Jae Kim, Ohgew Kweon

**Affiliations:** 1 Division of Bioinformatics and Biostatistics, National Center for Toxicological Research, U.S. Food and Drug Administration, Jefferson, AR 72079, USA; jaehyun.kim@fda.hhs.gov; 2 Division of Microbiology, National Center for Toxicological Research, U.S. Food and Drug Administration, Jefferson, AR 72079, USA; sunghyun.yoon@fda.hhs.gov (S.Y.); sandeep.kondakala@fda.hhs.gov (S.K.); steven.foley@fda.hhs.gov (S.L.F.); mark.hart@fda.hhs.gov (M.H.); jbsnfrs@earthlink.net (J.B.S.); 3 Department of Oral Microbiology and Immunology, School of Dentistry, Dankook University, Cheonan 31116, Republic of Korea; micro94@dankook.ac.kr; 4 Department of Management, Marketing, and Technology, University of Arkansas at Little Rock, Little Rock, AR 72204, USA; wwang@ualr.edu (W.W.); sxkim@ualr.edu (S.-K.K.)

**Keywords:** prokaryote, genome sequencing, complete genome, metadata, genomic features, taxonomy, dashboard

## Abstract

Prokaryotes, the earliest forms of life on Earth, play crucial roles in global biogeochemical processes in virtually all ecosystems. The ever-increasing amount of prokaryotic genome sequencing data provides a wealth of information to examine fundamental and applied questions through systematic genome comparison. Genomic features, such as genome size and GC content, and taxonomy-centric genomic features of complete prokaryotic genomes (CPGs) are crucial for various fields of microbial research and education, yet they are often overlooked. Additionally, creating systematically curated datasets that align with research concerns is an essential yet challenging task for wet-lab researchers. In this study, we introduce CPGminer, a user-friendly tool that allows researchers to quickly and easily examine the genomic features and taxonomy of CPGs and curate genome datasets. We also provide several examples to demonstrate its practical utility in addressing descriptive questions.

## 1. Introduction

**Figure 1:**
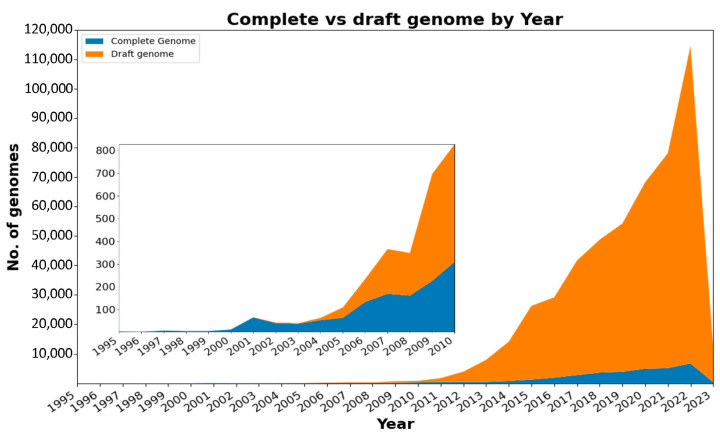
Complete and draft genomes submitted to NCBI by year.

Prokaryotes, the earliest forms of life on Earth, have been present for 4 billion years and play crucial roles in global biogeochemical processes in virtually all ecosystems [1]. The advent of genome sequencing technologies enabled the rapid and comprehensive characterization of prokaryotic genomes. Since the complete sequencing of the first prokaryotic genomes, those of the bacterium *Haemophilus influenzae* in 1995 [2] and the archaeon *Methanococcus jannaschii* in 1996 [3], the number of prokaryotic genome sequences publicly available has grown exponentially (Figure 1) [4,5]. We are now in the postgenomic era, where genome sequencing is a standard procedure and the increasing quantity and diversity of generated sequences have significant implications for data storage and analysis [6].

Prokaryotic genomes provide both sequence data (i.e., nucleotide and protein sequences) and metadata, including genomic features such as genome size, GC content, and the number of genes/proteins, as well as taxonomic information [5]. Complete prokaryotic genomes (CPGs) are gap-free sequences of prokaryotic genomes. They can provide a more robust examination of fundamental questions, including taxonomy, phylogeny, and genomic dynamics. Further, CPGs possess significant practical applications, providing substantial contributions to genome-scale metabolic modeling, enhancing the scope of biosurveillance, supporting bioforensics, and facilitating the study of infectious disease epidemiology [7,8,9].

The efficacy of comparative genomics can be markedly enhanced with the availability of more complete genomes, relevant metadata regarding the sequenced specimen and its genome, and advanced bioinformatics tools for downstream analyses [10]. Concurrently, constructing curated genome datasets pertinent to research objectives is imperative, despite being a demanding task for many laboratory scientists. Various web-based biological databases and portals, which are publicly accessible and possess wide-ranging functionalities, cater to the prerequisites for successful genome comparison studies [11]. However, the taxonomy of CPGs and the correlation between genomic attributes, such as genome size and GC content, are a crucial yet frequently neglected piece of information vital to microbiological research and education. To address these gaps, we developed CPGminer, a user-friendly, web-based, interactive dashboard that enables users to rapidly and accurately estimate genomic features of CPGs and also to curate genome datasets based on genomic features and taxonomy.

## 2. Materials and Methods

### 2.1. Programming Language and Library

CPGminer was developed using Streamlit, an open-source Python library that can be used to easily create and share interactive web applications [12,13]. CPGminer (its source code and an executable file [CPGminer.exe]) is available in a public GitHub repository (https://github.com/jayprimer/CPGminer, accessed on 22 September 2023).

### 2.2. Customized CPG Metadata

The metadata of partial and complete prokaryotic genomes is hosted at the National Center for Biotechnology Information (NCBI, https://ftp.ncbi.nlm.nih.gov/genomes/GENOME_REPORTS/prokaryotes.txt, accessed on 22 September 2023). This information is cached daily by CPGminer to generate customized CPG metadata, including genomic features (e.g., genome size, GC%, and the number of chromosomes, plasmids, genes, and proteins) and taxonomic information. CPGminer counts the numbers of chromosomes and plasmids in each CPG by parsing the “replicon” information of the original metadata. For the taxonomic-ranks-based search/filter function, CPGminer uses the ETE Toolkit (ETE 3 Python library, http://etetoolkit.org/, accessed on 22 September 2023) to generate taxonomic information (from superkingdom to species) from the “taxID” of each CPG [14]. Finally, CPGminer provides users with download links for data tables and figures. The customized CPG metadata comprises 17 metadata fields, which are organized into two categories: taxonomy and genomic features.

### 2.3. User Interface

**Figure 2:**
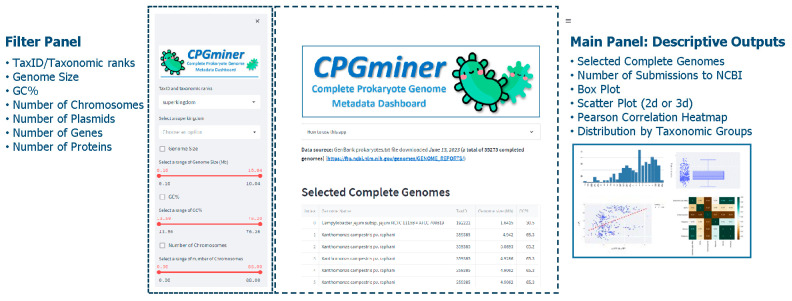
Overview of CPGminer.

CPGminer is a web-based tool that allows users to search and filter through a large collection of complete prokaryotic genome (CPG) metadata. The tool’s interactive online interface provides a user-friendly two-column layout with a sidebar on the left for filtering data and a main panel for tables and charts with selected data (Figure 2).

The filter panel in CPGminer offers several parameters for users to select from. Users can search and filter by taxonomic ranks and general genomic features. Taxonomic ranks are selected using two drop-down boxes, while general genomic features such as genome size, GC%, number of chromosomes, plasmids, genes, and proteins are selected using checkboxes and sliders. Users can also select a combination of taxonomic ranks and genomic features for a more detailed and granular selection of CPGs.

As users select search terms and adjust filter values, the main information panel displays predefined tables and figures for the selected CPGs as follows:

- Selected complete genomes: A downloadable table of selected genomes with 17 metadata fields including genome name, taxID, genome size, GC%, replicons, numbers of chromosomes, plasmids, genes, and proteins, release date, genome download FTP address, and taxonomic information fields from superkingdom to species. This table serves as underlying data for the subsequent plots.
- Box plot for genomic features: genome size, GC%, and numbers of chromosomes, plasmids, genes, and proteins.
- Number of submissions to NCBI by year.
- Scatter plot for genomic features: Users can select two or three genomic features and it will display a 2D or 3D scatter plot depending on the number of genomic features users select.
- Pearson correlation heatmap of genomic features.
- Distribution by taxonomic groups: A downloadable table of selected genomes that shows their count in each taxonomical grouping. Users can select one level from superkingdom to species and the resulting table displays all available taxonomical categories to the selected level and its count.

## 3. Results and Discussion

CPGminer provides a web-based, graphical user interface to rapidly search, filter, visualize, save, and download matching CPG metadata. In the following, we demonstrate how CPGminer can be used in different scenarios by utilizing both genomic features and taxonomic information to address several descriptive questions.

### 3.1. Question 1: What Are the Median Values of Genome Size and Guanine + Cytosine (GC) Content of Bacterial Genomes and the Correlation between Them?

Bacteria possess a wide range of genome sizes and GC contents, reflecting their unique adaptations to different environments [15,16]. An overarching trend observed across bacterial genomes is that there is a correlation between genome size, GC content, environmental niches, and lifestyle among bacterial genomes [15,16].

**Figure 3:**
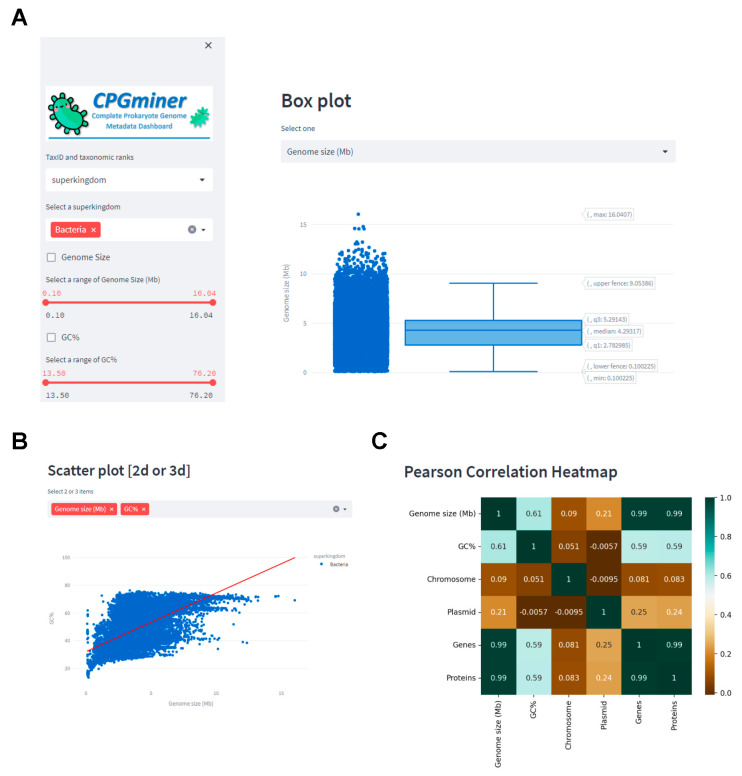
Genome size and GC content of CPGs. (**A**) CPGminer showing a box plot of genome sizes of CBGs. (**B**) Scatter plot of genome size (Mb) and GC% of CBGs. (**C**) Heatmap showing correlation among the genomic features, including genome size and GC%.

To address the question, CPGminer was used to access daily updated metadata of CPGs (Figure 3). To select only complete bacterial genomes (CBGs), the “superkingdom” option was chosen from the “TaxID and taxonomic ranks” drop-down menu, and “Bacteria” was selected from the subsequent “Select a superkingdom” drop-down menu. To obtain the median values of genome size and GC content of bacteria, “genome size” or “GC%” were chosen from the “Boxplot data of genome size, No. of chromosomes, plasmids, genes, and proteins, and GC%” drop-down menu in the “Descriptive statistics” section (Figure 3A).

As of 11 May 2023, a total of 32,143 CBGs, representing 60 phyla, 1717 genera, and 7084 species, were selected. The median genome size and GC content for these CBGs were 4.24 Mb and 50.7%, respectively. However, there was a wide range in the data, with genome sizes ranging from 0.11 Mb to 16.04 Mb and GC contents ranging from 13.5% to 75.6%. The largest genome (as of 11 May 2023) in GenBank is that of *Minicystis rosea* DSM 24000, with a size of 16.04 Mb and 14,117 genes (https://www.ncbi.nlm.nih.gov/genomes/all/GCF/001/931/535/GCF_001931535.1_ASM193153v1/, accessed on 11 May 2023). On the other hand, the smallest bacterial genome sequenced is that of *Candidatus Nasuia deltocephalinicola* PUNC, with a size of only 0.112 Mb and 173 genes (141 proteins) (https://ftp.ncbi.nlm.nih.gov/genomes/all/ GCF/001/447/885/GCF_001447885.1_ ASM144788v1/, accessed on 11 May 2023). The correlation coefficient (r-value) between genome size and GC content for CBGs was 0.61, indicating a strong correlation between the two features in bacteria.

### 3.2. Question 2: Which Phyla, Genera, and Species of Bacteria Represent the Top Five Complete Genome Sequences?

Since the first bacterial genome (i.e., *H. influenzae*) was completely sequenced in 1995 [2], over 500,000 complete or draft prokaryote genomes have been uploaded to the NCBI database (including 32,603 complete genomes as of 11 May 2023) (https://www.ncbi.nlm.nih.gov/genome/browse#!/prokaryotes/, accessed on 10 October 2023). Although metagenome-assembled genomes (MAGs) from metagenomics data are not limited by culturability, most of the prokaryote genomes in the NCBI database are from pure cultures [17]. Therefore, the current statistics of sequenced prokaryotic genomes seem to be a clue to see microbiological research trends, including diversity in researchers’ attention, research subjects, and prokaryotic culturability [4].

To obtain the distributions of CBGs at three different taxonomic ranks, we first selected “superkingdom” from the select box “TaxID and taxonomic ranks” and then chose “Bacteria” from the select box “Select a superkingdom” to select CBGs only. We then selected “Phylum”, “Genus”, or “Species” from the select box in the section “Distribution by Taxonomic Groups” to download the distribution data of the selected complete genomes in CSV file format.

**Figure 4:**
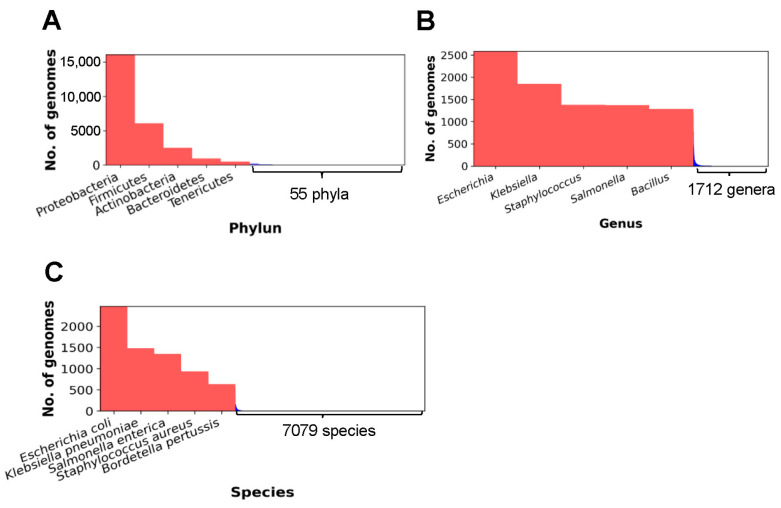
Top five bacterial phyla (**A**), genera (**B**), and species (**C**) with the highest number of CPGs.

As shown in Figure 4, 32,143 CBGs (NCBI, May 2023) are from 60 phyla, 1717 genera, and 7084 species. Among the 60 phyla, Proteobacteria, Firmicutes, Actinobacteria, Bacteroidetes, and Tenericutes are the top five for the number of sequenced genomes, with 18,876, 7315, 2839, 1146, and 574, respectively (Figure 4A). At the genus level, *Escherichia*, *Klebsiella*, *Staphylococcus*, *Salmonella*, and *Bacillus* are the top five genera with sequenced genome numbers of >1000 (Figure 4B). On the other hand, among the 7084 bacterial species, *E. coli*, *K. pneumoniae*, *S. enterica*, *S. aureus*, and *Bordetella pertussis* are the top five species with completely sequenced genomes from 639 to 2488 (Figure 4C).

### 3.3. Question 3: What Percentage of Prokaryotes Have Multiple Chromosomes? What Percentage of Prokaryotes Have Plasmid(s)?

There was a long-held paradigm that the bacterial genome comprises a single circular chromosome and additional dispensable nonessential plasmid(s). However, it is now clear that some bacteria have multiple chromosomes [18,19]. Jha et al. (2012) reported that about 10% of sequenced bacteria have split genomes, and this estimate was quoted repeatedly [19,20,21,22,23].

In order to scrutinize the estimate, we used CPGminer to count the number of multichromosomal prokaryotes in the NCBI database. To select only multichromosomal prokaryotes, we selected the checkbox “Number of Chromosome” from the left search/filter panel and then set the range of the slide bar “No. of Chromosome” to ≥2. On the other hand, in order to calculate the ratio between prokaryotes with one or more plasmids and prokaryotes without a plasmid, we obtained the number of prokaryotes with one or more plasmids by selecting the checkbox “No. of Plasmid” and then set the range of the slide bar “No. of Plasmid” to ≥1.

Our estimation indicates that about 5.0% (1633 out of 32,603) of CPGs in the NCBI database are multichromosomal. About 5.0% (1628 out of 32,143) of CBGs have two or more chromosomes, whereas only 1.1% (5 out of 460) of complete archaeal genomes (CAGs) have two chromosomes. The multipartite genomes are scattered throughout about 77 prokaryotic genera, including *Vibrio*, *Burkholderia*, *Brucella*, *Leptospira*, *Agrobacterium*, and *Prevotella*. On the other hand, approximately 43.8% (14,279/32,603) of completely genome-sequenced prokaryotes contain one or more plasmids: 44.0% (14,156/32,143) and 26.7% (123/460) for bacteria and archaea, respectively. Interestingly, although single-chromosomal prokaryotes show a higher rate of the presence of plasmids than multichromosomal prokaryotes, there is no statistical significance (X2 [1, N = 32,603] = 0.68, *p* = 0.40713).

## 4. Conclusions

CPGminer is a user-friendly, web-based, interactive dashboard that enables users to systematically access, explore, and analyze CPG metadata, which is essential for various biological research and education activities. Additionally, CPGminer has the potential for further improvement in terms of content and functionality to meet the evolving needs of researchers in the field.

## Data Availability

CPGminer is available in a public GitHub repository (https://github.com/jayprimer/CPGminer, accessed on 22 September 2023).
